# Supplementary material for: Remediation and upcycling of microplastics by algae with wastewater nutrient removal and bioproduction potential
Source: Nat Commun. 2025 Dec 22;16:11570. doi: 10.1038/s41467-025-67543-5 (PMC12748894; doi:10.1038/s41467-025-67543-5)
Supplement: Supplementary file 1 — Supplementary Information [file 41467_2025_67543_MOESM1_ESM.pdf]

Remediation and upcycling of microplastics by algae with wastewater nutrient removal and bioproduction potential

Bin Long[1,2,3,4#], Qiang Li[1,2#], Cheng Hu[1,2,3,4], Yayun Chen[5], Yining Zeng[6], Weiwei Li[7], Sydney Pearson[1,2], Mengqiao Liu[5], Chengcheng Fei[5], Joshua S. Yuan[1,2,7*], Susie Y. Dai[1,2,3,4*]

[1] Department of Plant Pathology and Microbiology, Texas A&M University, College Station, TX 77843, USA

[2] Synthetic and Systems Biology Innovation Hub (SSBiH), Texas A&M University, College Station, TX 77843, USA

[3] Department of Chemical and Biomedical Engineering, University of Missouri, Columbia, MO 65201, USA

[4] Christopher S. Bond Life Science Center, University of Missouri, Columbia, MO 65211, USA

[5] Department of Agricultural Economics, Texas A&M University, College Station, TX 77843, USA

[6] Renewable Resources and Enabling Sciences Center, National Renewable Energy Laboratory, Golden, CO, 80401, USA

[7] Department of Energy, Environmental, and Chemical Engineering, Washington University in St. Louis, St. Louis, MO 63130, USA

[#]These authors contributed equally: Bin Long and Qiang Li.

*Corresponding contributor. E-mail: *joshua.yuan@wustl.edu* *sydai@missouri.edu*

Supplementary Figures

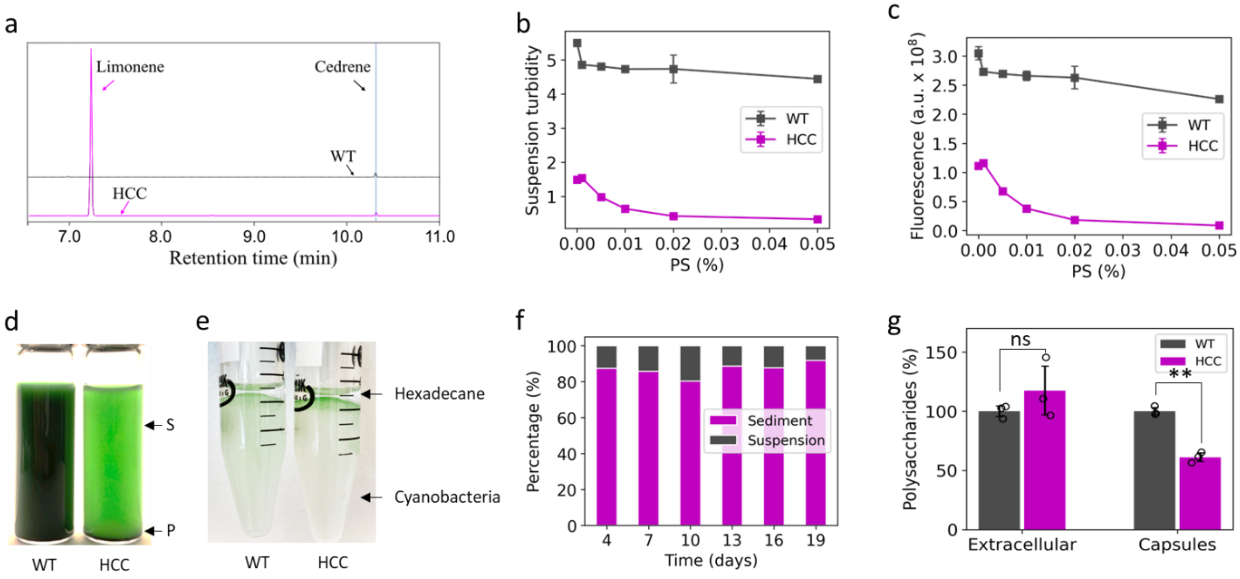

Supplementary Figure 1. Characterization of HCC and HCC-mediated microplastic removal. (a) Limonene production was detected in the HCC strain but not in the WT strain, as analyzed by GC-MS, with cedrene used as an internal control. (b) Suspension turbidity of HCC-PS samples was significantly lower ($p < 0.01$) than that of WT-PS samples across all tested PS concentrations. (c) Chlorophyll fluorescence of cyanobacteria was significantly lower ($p < 0.01$) in HCC-PS suspensions compared to WT-PS suspensions. (d) Cell aggregation and auto-sedimentation were observed in HCC samples but not in WT samples. "S" represents suspension, and "P" represents pellet. (e) The BATH assay showed that more HCC cells attached to the hydrophobic hexadecane layer, reducing the color in the aqueous layer, which indicates increased hydrophobicity of HCC cells. (f) Percentages of dry weight in suspension and sediment during HCC-based long-term microplastic removal. Auto-sedimentation remained effective even after 19 days of semi-continuous cultivation. (g) Polysaccharide measurements estimating EPS content in HCC and WT samples. While no significant difference ($p = 0.3$) was observed in extracellular polysaccharides, WT samples exhibited significantly higher ($p = 0.0003$) capsular

polysaccharides. Data are presented as mean values ± standard deviations (n = 3 independent samples). ** indicates $p < 0.01$. ns indicates no significance. Two-tailed Student's t-test was used. Source data is provided as a Source Data file.

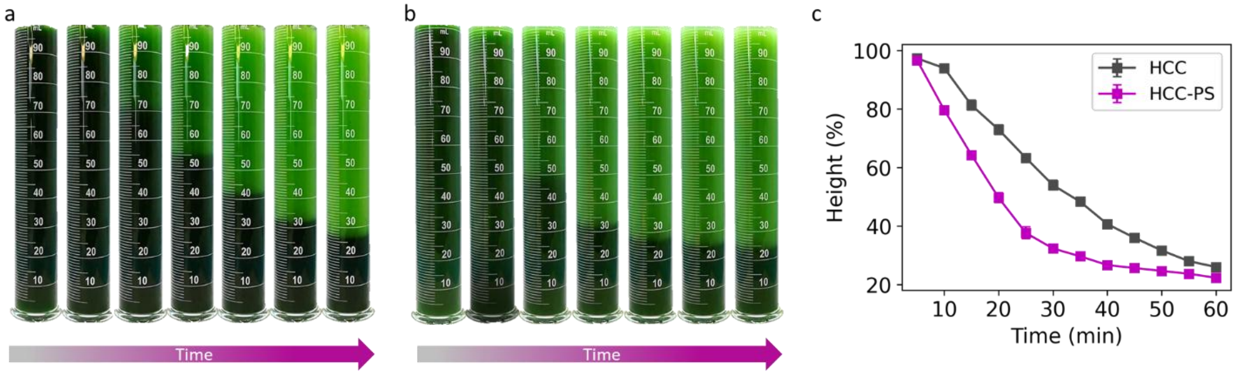

Supplementary Figure 2. Sedimentation was accelerated by adding polystyrene (PS) microplastics. (a) sedimentation without addition of polystyrene microplastics. (b) sedimentation with 0.05% (w/v) polystyrene microplastics. Time points from left to right are 0, 10, 20, 30, 40, 50, and 60 min after sedimentation. (c) quantification of sedimentation with /without polystyrene. To determine the height of sedimentation, cyanobacterial cells with /without polystyrene were allowed to settle in a graduated cylinder, and the height was measured by recording the position of the upper edge of the dark green sediment layer against the cylinder's scale. Significant differences ($p < 0.05$) were observed at time points of 10, 20, 30, 40, and 50 mins. Two-tailed Student's t-test was used. Source data is provided as a Source Data file.

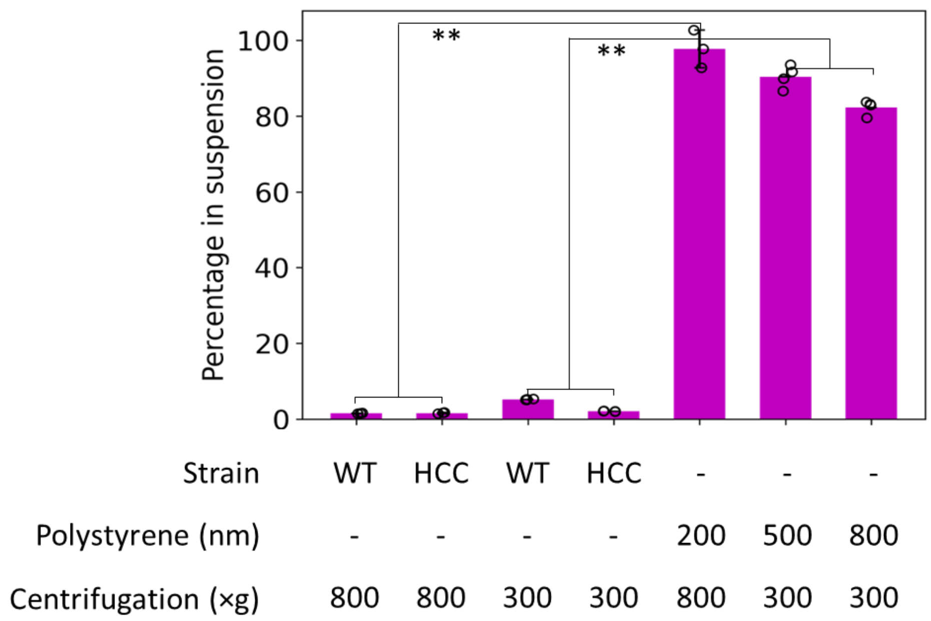

Supplementary Figure 3. Evaluation of the separation of PS microplastics and cyanobacterial cells using low-speed centrifugation. The mixture of cyanobacterial cells and 200 nm polystyrene microplastics was separated by centrifugation at 800 ×g for 3 minutes. Under these conditions, nearly all cyanobacterial cells (with only about 1.5% of WT and HCC cells remaining in suspension) were pelleted, while the majority of 200 nm polystyrene microplastics (97.9%, $p < 0.01$) remained suspended. For mixtures containing 500 nm and 800 nm polystyrene microplastics, centrifugation was performed at 300 ×g for 3 minutes. In this case, 5.2% of WT cells and 2.0% of HCC cells remained in suspension, whereas most of the polystyrene microplastics - 90.4% of the 500 nm and 82.3% of the 800 nm particles - remained suspended ($p < 0.01$). These results demonstrate that low-speed centrifugation is an effective method for separating and estimating the content of cell-microplastic mixtures. Data are presented as mean values ± standard deviations (n = 3 independent samples for 800 ×g and n= 4 independent samples for 300 ×g). Two-tailed Student's t-test was used. Source data are provided as a Source Data file.

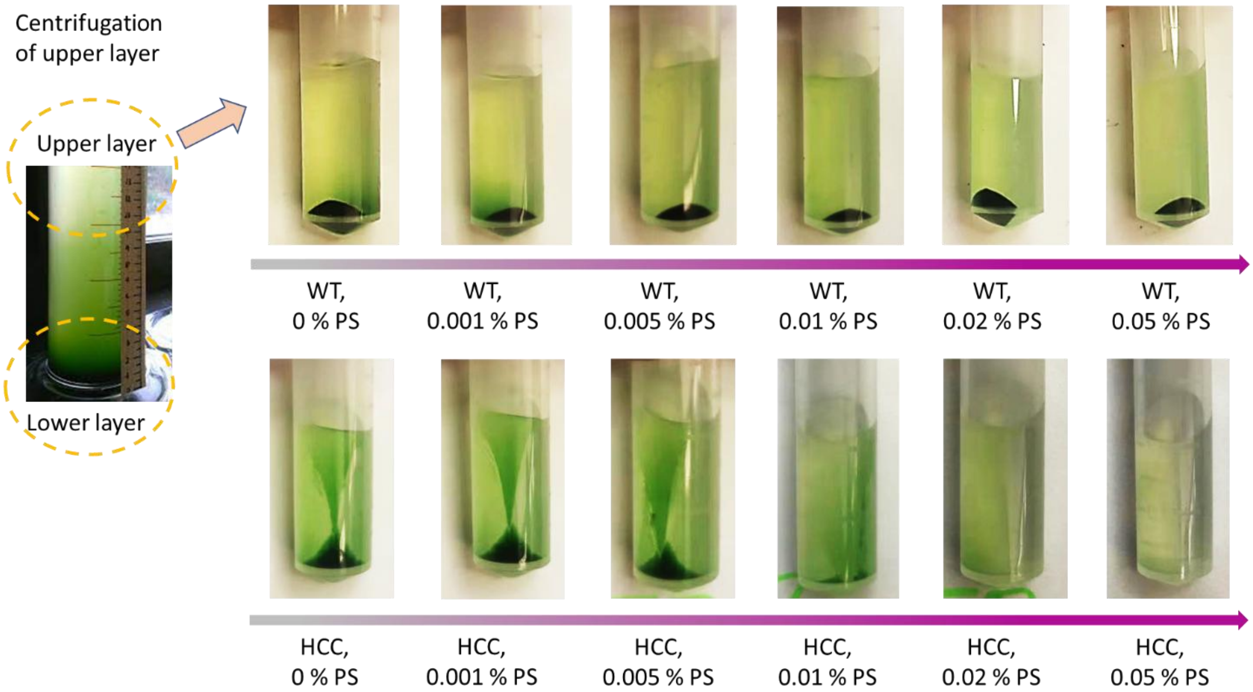

Supplementary Figure 4. Solid content visualization of suspensions in the WT and HCC treated samples. After microplastic removal by sedimentation, the suspension samples (upper layer as indicated in the figure) were centrifuged to visualize the solid contents in the suspension samples. The solid contents were higher in the WT samples than those in the HCC samples. Additionally, HCC samples with higher polystyrene concentrations exhibited lower solid content compared to those with lower concentrations. The experiment was repeated three times and similar results were obtained.

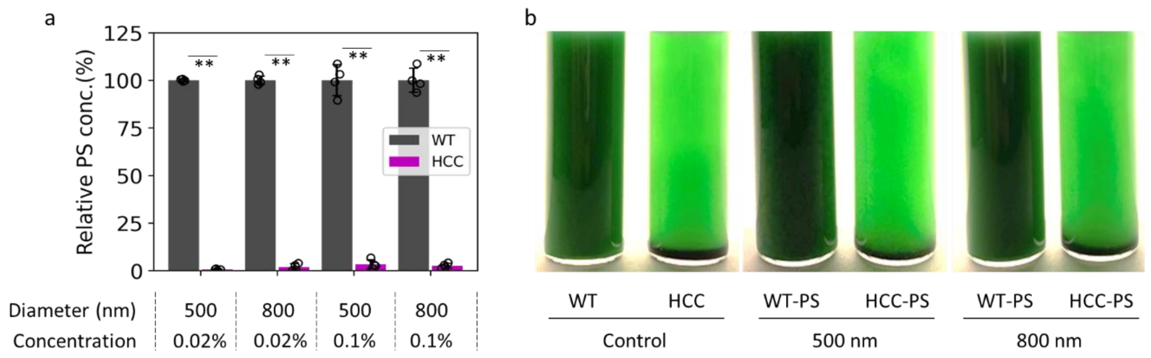

Supplementary Figure 5. Microplastic removal tests for 500 nm and 800 nm polystyrene microplastics. a. Significantly ($p < 0.01$) higher microplastic removal rate was observed in the HCC samples compared to the WT samples. The suspension turbidity after low-speed centrifugation of WT samples for each treatment was normalized to 100%. The turbidity of the centrifuged suspension in the HCC samples accounted for only 0.6% and 1.8% of that in the WT samples when 0.02% of 500 nm and 800 nm polystyrene microplastics were applied, respectively. These values slightly increased to 3.3% and 2.5% when 0.1% of 500 nm and 800 nm polystyrene microplastics were applied, respectively. b. Sedimentation was observed in the HCC samples, but not in the WT samples, regardless of the presence of microplastics, as indicated by the accumulation of sediments at the bottom of the sample and the lighter color of the suspension. The experiment was repeated three times and similar results were obtained. ** indicates $p < 0.01$. Two-tailed Student's t-test was used. Source data are provided as a Source Data file.

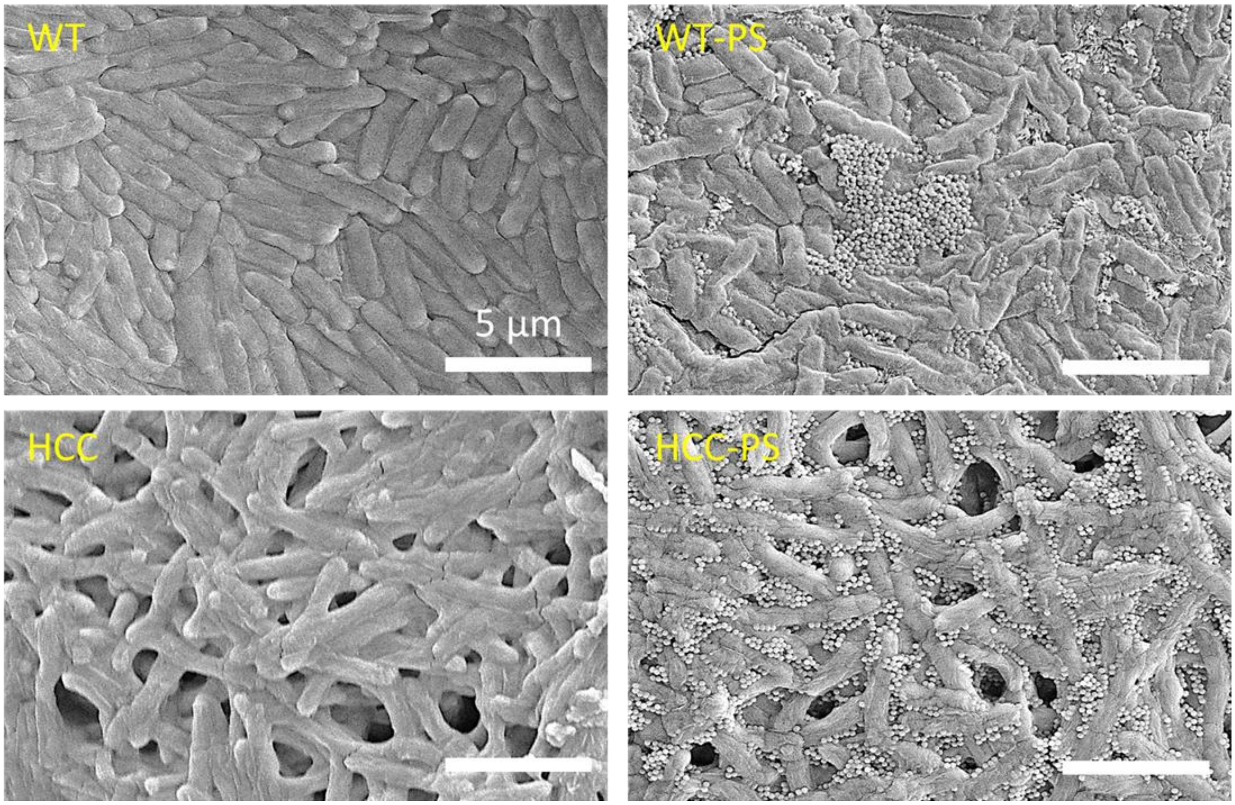

Supplementary Figure 6. SEM images showing the interactions between polystyrene microplastics and cyanobacterial cells. In the WT-PS sample, the microplastics exhibited random attachment to WT cells. In the HCC sample, a more uniform attachment of polystyrene microplastics to HCC cells was observed, and the attachments are primarily located at cell intersections. These results highlight the consistent interactions between polystyrene and HCC.

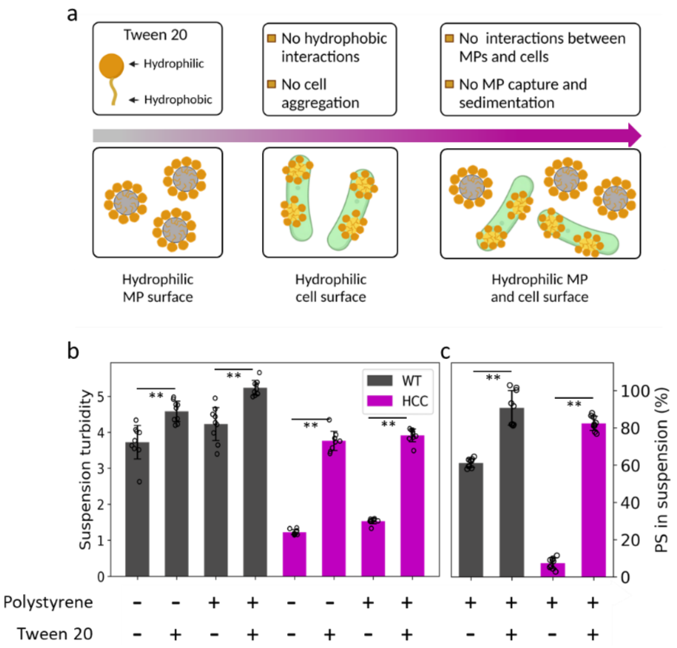

Supplementary Figure 7. Surfactant blocking to verify the hydrophobic interactions. Tween 20, a surfactant consisting of a hydrophilic head and a hydrophobic tail, was used to block the hydrophobic surfaces of both cyanobacteria and microplastics (a). When added to the samples, the hydrophobic tails of Tween 20 molecules interacted with the hydrophobic interfaces of the cyanobacterial cells and microplastics (MPs), exposing their hydrophilic heads to the solution (a). This was expected to inhibit cell-to-cell and cell-to-microplastic aggregations, which was supported by the significantly higher suspension turbidities observed when Tween 20 was added to the samples (b). Further analysis of polystyrene abundance in suspension (after low-speed centrifugation) also validated that Tween 20 substantially blocked microplastic removal (c). These results suggest that the hydrophobicity effect is the driving force for cell sedimentation and microplastic removal. Data are presented as mean values ±standard deviations (n = 9 independent samples). ** indicate $p < 0.01$. The symbols "+" and "–" indicate the presence or absence, respectively, of the specified components in the treatments. Two-tailed Student's t-test was used. Source data is provided as a Source Data file.

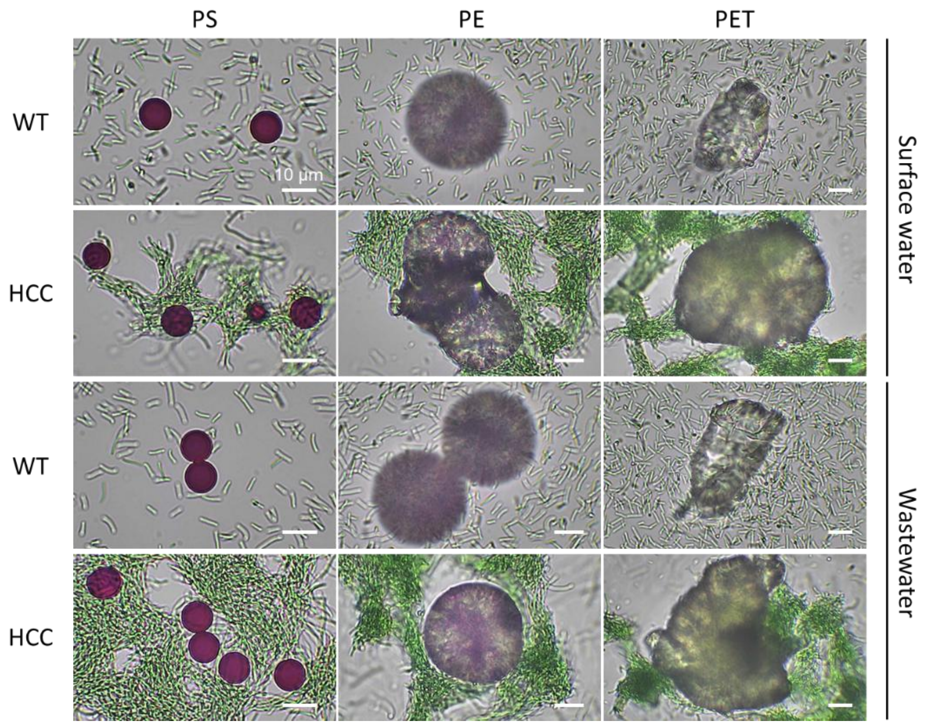

Supplementary Figure 8. HCC interacts with microplastics spiked in surface water and wastewater. Polystyrene (PS), polyethylene (PE), and polyethylene terephthalate (PET) microplastics were introduced into surface water and wastewater. Interactions between HCC cells and microplastics were observed in both environments, whereas no such interactions were detected between WT cells and microplastics.

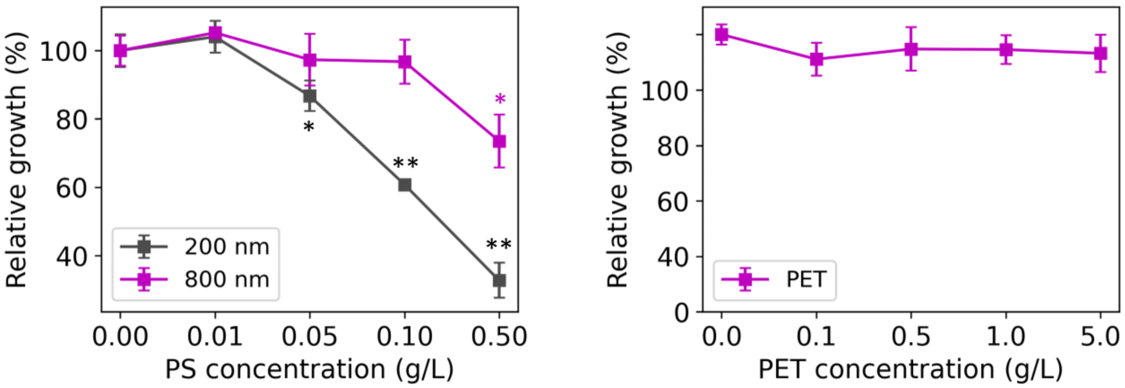

Supplementary Figure 9. Evaluation of cyanobacteria growth inhibition by microplastics. Polystyrene (PS) microplastics with diameters of 200 nm and 800 nm, as well as PET microplastics (<300 µm), were each added to cyanobacterial cells during cultivation. The cells were cultivated under illumination of 150 µmol/m²/s at a temperature of 39 °C, with a shaking speed of 400 rpm, in a CO_2 chamber set to 1% CO_2 for 2 days. The growth of cyanobacteria was estimated using chlorophyll fluorescence (Ex 595 nm and Em 690 nm). The chlorophyll fluorescence of the control group (without microplastic addition) was normalized to 100%. The results showed that at low concentrations, microplastics had marginal impacts on cyanobacterial growth. As the concentration increased, both 200 nm and 800 nm microplastics negatively affected cyanobacterial growth, with the smaller diameter microplastics having a stronger inhibitory effect. No significant growth inhibition was observed at any concentration tested for PET microplastics, presumably due to their larger size, which causes them to settle at the bottom of the growth container, resulting in less contact with the cyanobacterial cells and reduced shading on cyanobacterial cells. Data are presented as mean values ±standard deviations (n = 3 independent samples with three technical replicates). * indicates $p < 0.05$. ** indicates $p < 0.01$. Two-tailed Student's t-test was used. Source data is provided as a Source Data file.

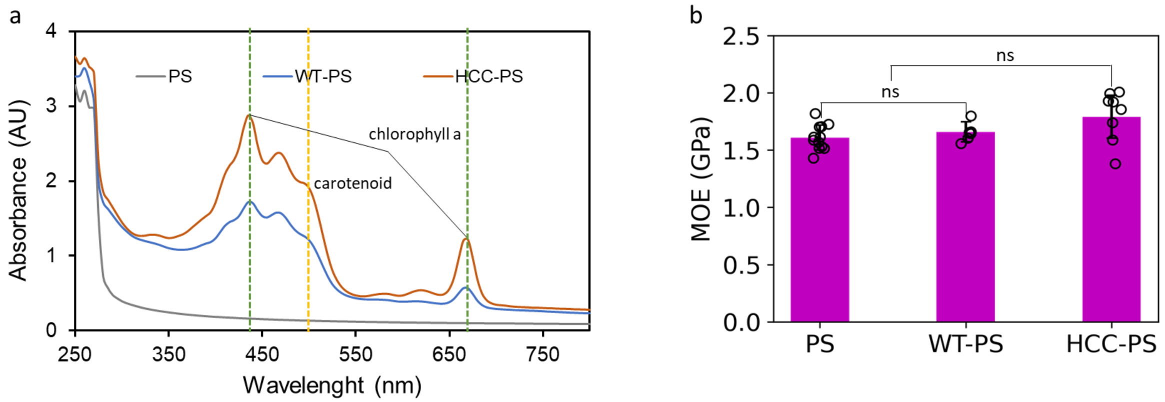

Supplementary Figure 10. Characterization of the upcycled bioplastics. (a) Absorbance of UV-Vis spectrum of PS and bioplastics. The absorbance peaks of chlorophyll a and carotenoids were detected in bioplastic composites, suggesting that these pigments are present in bioplastic films and may be responsible for their "golden green" color. The experiment was repeated at least three times and similar results were obtained. (b) Modulus of elasticity comparison. No significant differences ($p > 0.05$) were observed between the polystyrene (PS) plastics and bioplastics made from WT-PS or HCC-PS samples, in terms of modulus of elasticity. Data is presented as mean values ±standard deviations (n = 12 independent samples for PS, 6 independent samples for WT-PS, and 8 independent samples for HCC-PS). Two-tailed Student's t-test was used. Source data is provided as a Source Data file.

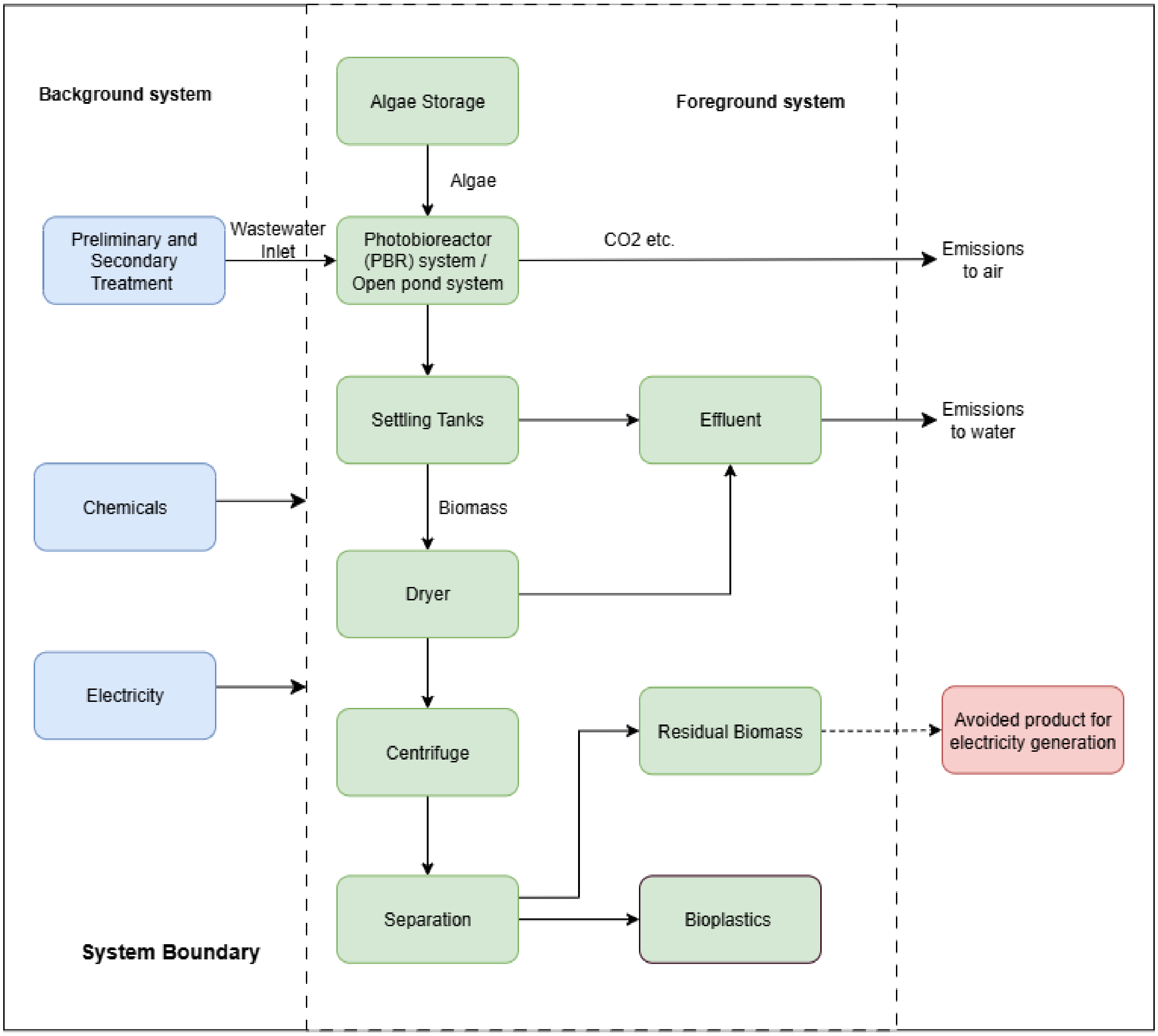

Supplementary Figure 11. **The boundary of the RUMBA-based biomass production, wastewater treatment, microplastic removal and upcycling system.** The background data are referenced from Ecoinvent 3.9, which includes the upstream manufacturing processes, chemical, electricity, and wastewater treatment inventory data. The foreground system comprises algae cultivation, biomass recovery, and biomass separation for bioplastic production, as determined by experimental data. The short-term algae storage stage is assumed to be negligible, consistent with the practice 1) general LCA analysis on algal biofuel application system does not take account of

CO_2 emission[1–3], 2) an emission estimate of an open pond system that operates for months long durations[4,5]. Our algal storage is intended for about three days, which resulted in about 0.0006g CO_2-eq/L wastewater, which is negligible to the total CO_2 emission. For system expansion, we assumed that the residual biomass can be used for electricity generation and sold to the grid.

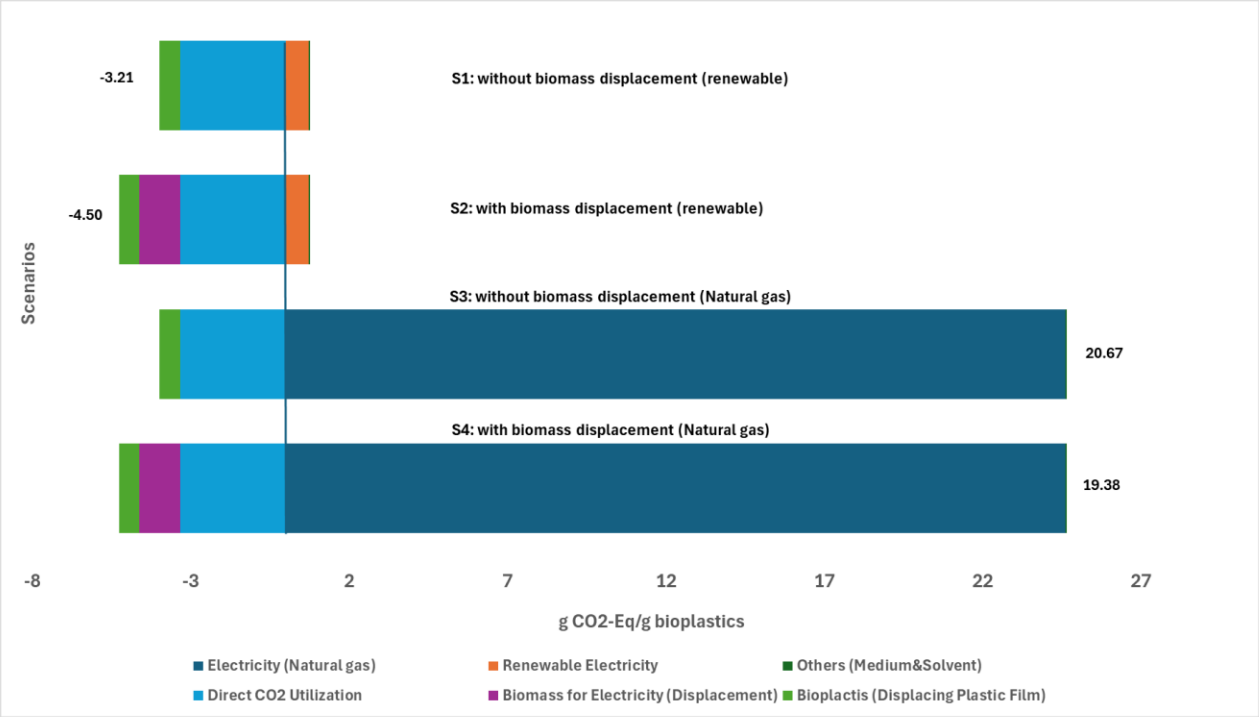

Supplementary Figure 12. Scenario analysis for RUMBA-based biomass production, wastewater treatment, microplastic removal and upcycling system. Scenarios 1 and 2 assumed the system used electricity from the U.S. renewable energy (wind onshore source in Texas) power plants. Scenario 1 assumes no residual biomass is used for electricity generation. Scenario 2 assumes the residual biomass is used for electricity generation. Others include emissions from medium, solvent, and wastewater treatment plant plastic removal. Scenarios 3 and 4 assumed the system used U.S. conventional electricity with/without byproducts displacement. Source data is provided as a Source Data file.

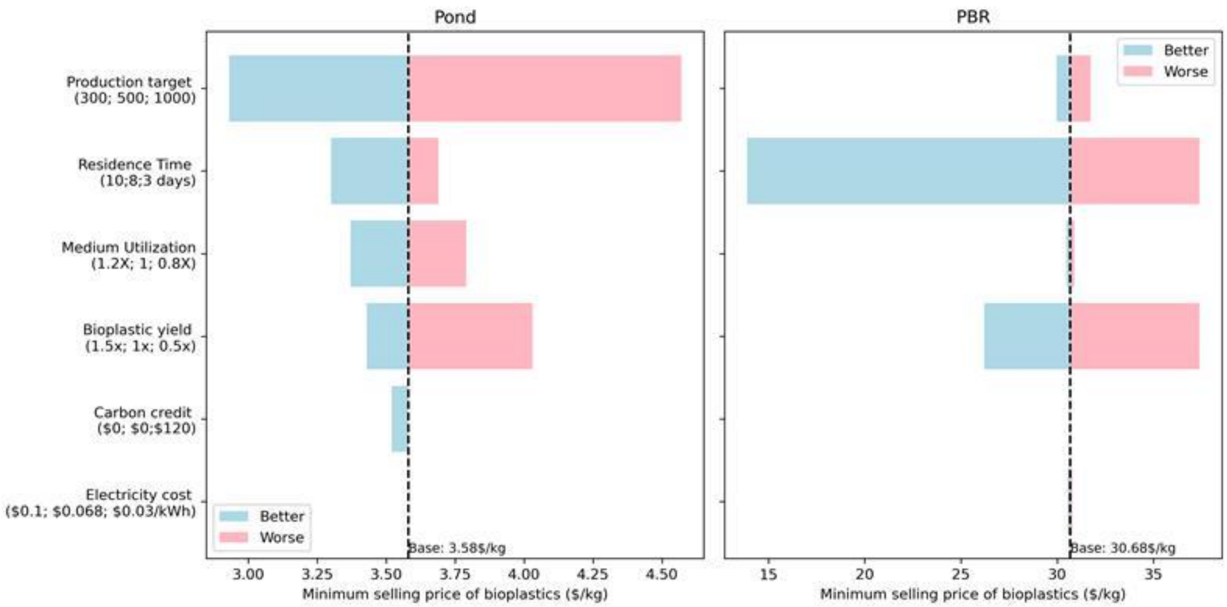

Supplementary Figure 13. Sensitivity analysis for the minimum selling price of bioplastics ($/kg) based on RUMBA-based microplastic upcycling system. The baseline scenario (vertical line) was calculated using the second number in each label as the input data, which results in a minimal selling price of bioplastics at $3.58/kg for the open pond system and $30.68/kg for the photobioreactor (PBR) system. The better and worse scenario analysis was then made, assuming the current analyzed parameter takes the better assumption (the first number in the label) or the worse assumption (the third number in the label), but all other parameters take the baseline assumptions (the second number in the label). The numeric results can be found in **Supplementary Table 6.** Source data is provided as a Source Data file.

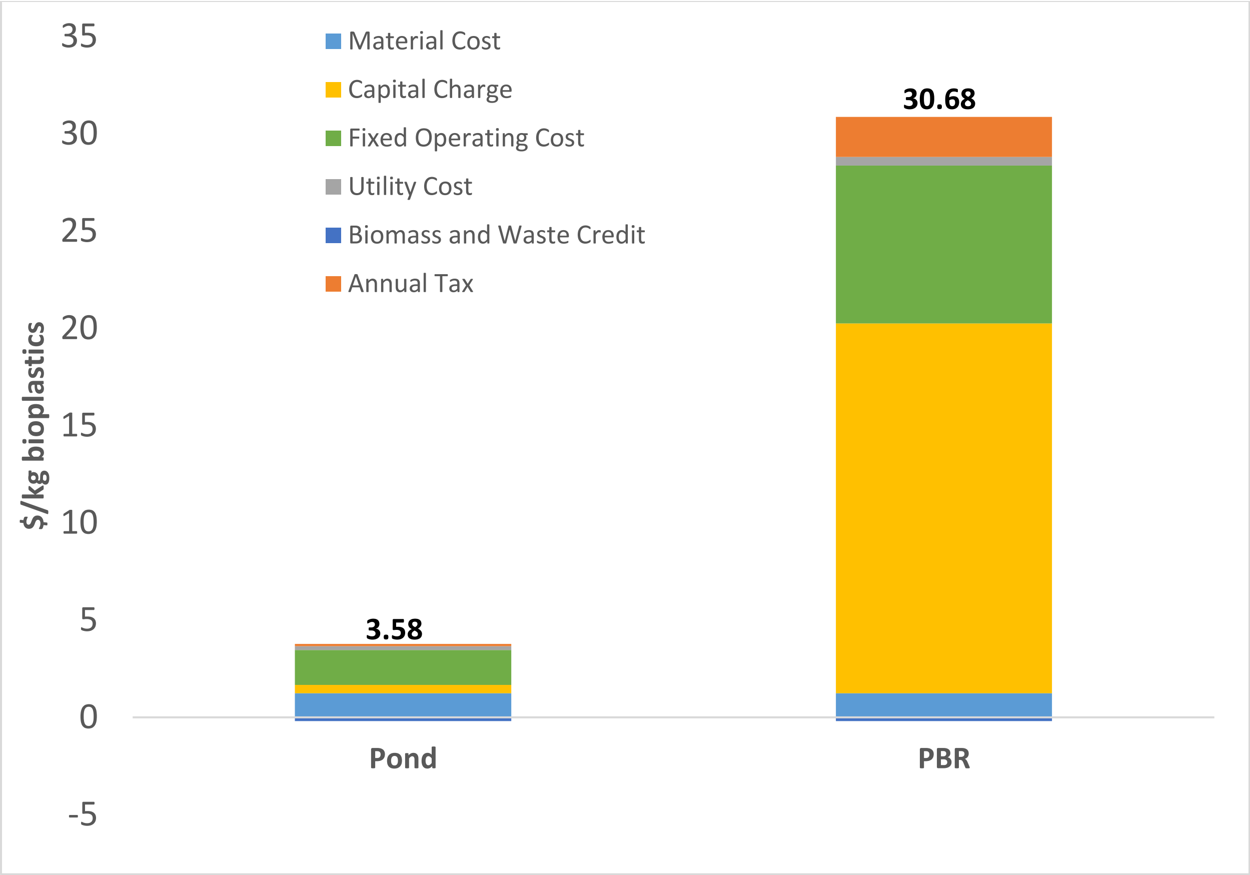

Supplementary Figure 14. Cost breakdown of minimum bioplastic selling price (MSP) for PBR and open pond system. The proportion of each type of cost in the MSP for both cultivation systems is calculated. Since the residual biomass is used for electricity generation and adds revenue for the system, it is represented as a negative value in the cost breakdown. The primary cost in the pond system is derived from raw material purchases, whereas the primary cost in the photobioreactor (PBR) system is associated with equipment investment. Source data is provided as a Source Data file.

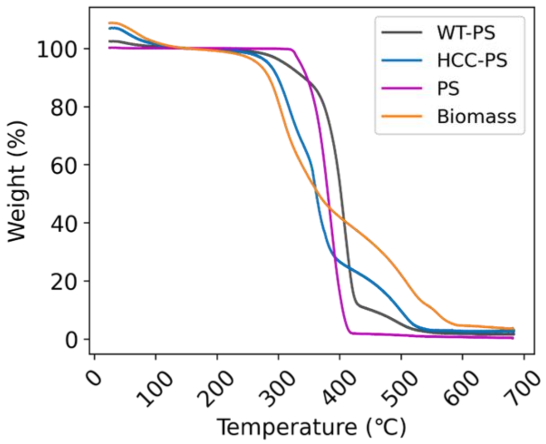

Supplementary Figure 15. Thermogravimetric analysis (TGA) results. TGA was used to measure the composition of suspension samples (WT-PS and HCC-PS), pure polystyrene (PS) microplastics, and cyanobacterial biomass (HCC). The experiment was repeated at least two times and similar results were obtained. Source data is provided as a Source Data file.

Supplementary Table 1. Wastewater Nutrient Removal by Cyanobacteria.

	Nitrate removal (%)	Ammonia removal (%)	Phosphate removal (%)
Influent	47.4 ± 0.6 (b)	100.0 (a)	34.6 ± 1.1 (b)
Effluent	97.5 ± 0.4 (a)	N.A.	37.8 ± 1.9 (b)
Influent + BG11	96.7 ± 0.1 (a)	100.0 (a)	99.8 ± 0.1 (a)
Effluent + BG11	96.4 ± 0.1 (a)	100.0 (a)	99.8 ± 0.1 (a)

Means followed by the same letter are not significantly different. For example, for all the results with (a), they are not different from one another. For results assigned with (a) and (b) separately, the results with (a) are different from the results assigned with (b).

Supplementary Table 2. Inputs and Outputs per Liter of Wastewater Treated

Process	S1 (S3)	S2 (S4)	Units
**Inputs**			
**Hydraulic Retention Time (HRT)**	8	8	days
**Water from Wastewater**	1.00	1.00	L
**N**	501	501	mg/L
**P**	20.95	20.95	mg/L
**Microplastic concentration**	0.50	0.50	g/L
**CO_2**	7	7	g/L
**Medium (excluding N&P source)**	See the medium table in Supplementary Table 3**Error! Reference source not found.**		
**Chloroform**	0.076	0.076	g/L
**Energy consumption**			
**PBRs**	0.00008	0.00008	kWh/L
**Centrifuge**	0.0014	0.0014	kWh/L
**Dryer**	0.076	0.076	kWh/L
**Separation**	0.0074	0.0074	kWh/L
**Total energy consumption**	0.085	0.085	kWh/L
**Outputs**			
**Emissions to water**			
**N**	6.54	6.54	mg/L
**P**	0.27	0.27	mg/L
**Microplastics**	0.11	0.11	g/L
**Emissions to the air**			
**CO_2**	1.90	1.90	g/L
**Bioplastics***	1.53	1.53	g/L
**Residual biomass**	2.66	2.66	g/L
**Avoided products**			
**Electricity from residual biomass****	/	0.0017	kWh/g
**Bioplastics for petroleum-based plastic film*****	1.53	1.53	g/L

Note: **Untreated wastewater is used as the baseline for comparison.** * With optimized strain selection, metabolic engineering, and cultivation under stress, microalgal systems could potentially reach ~50–70 wt%, although this remains largely unachieved in practice. **The electricity factor is 0.44368 kg CO_2-eq per kWh, also from Ecoinvent 3.9, for production from a natural gas combined-cycle power plant in Texas, USA (UUID: 8c40b198-fdcf-4ded-9def-ec647470107d). ***The emission factor is 0.65167 kg CO_2-eq per kg of plastic film (UUID: f5c54f00-b673-4a59-9df5-ef88a970cb20) from Ecoinvent 3.9. Assume a 1:1 functional

equivalence by bioplastic mass which is applicable to products such as agricultural mulch films, landscaping covers, construction sheets, and other applications[6,7].

Supplementary Table 3. Medium Components Exclude Nitrogen (N) and Phosphate (P) Sources

Medium Components	Usage (g)	Unit price ($/kg)	Cost ($/L wastewater)
$MgSO_4 \cdot 7H_2O$	7.50E-02	0.12	9.00E-06
$CaCl_2 \cdot 2H_2O$	3.60E-02	0.11	3.96E-06
Citric Acid$\cdot H_2O$	6.00E-03	0.89	5.34E-06
$FeCl_3$	3.64E-03	0.30	9.11E-07
$Na_2EDTA \cdot 2H_2O$	1.00E-03	2.00	2.00E-06
Na_2CO_3	2.00E-02	0.20	4.00E-06
$ZnSO_4$	2.20E-04	0.40	8.80E-08
$CuSO_4$	7.90E-05	0.10	7.90E-09
H_3BO_4	2.86E-03	1.05	3.00E-06
$MnCl_2 \cdot 4H_2O$	1.81E-03	2.00	3.62E-06
$Na_2MoO_4 \cdot 2H_2O$	3.90E-04	7.00	2.73E-06
$Co(NO_3)_2 \cdot 6H_2O$	4.94E-05	7.00	3.46E-07
		**Total**	**3.50E-05**

Note: This receipt is based on BG-11. The prices of the chemicals are quoted from Alibaba[8] (accessed March 2025).

Supplementary Table 4. Economic Assumptions for TEA Analysis

Parameter	Value
Target production level (Ton y^{-1})	500
Annual operating hours (h y^{-1})	7,920
Maintenance cost (US$ y^{-1})	1% of FCI
Plant lifetime (y)	30
Internal rate of return (%)[9]	10
Depreciation method	Modified Accelerated Cost Recovery System
Plant recovery period (y)	10
Construction period (y)	1
Lang factor	3
Start-up period (months)	6
Insurance (%)	0.8% of FCI
Local taxes (%)	2.5% of FCI
Income tax rate[9]	21%
Working capital cost	5 % of FCI

Note: FCI: fixed capital investment; the costs and prices are adjusted to the year 2024 based on the indices[10]. Wastewater treatment gains $0.02/m[9,11].

Supplementary Table 5. Equipment and Utility Costs

	**Purchase Cost Per Unit**	**Utility cost (USD/hr)**	**Electricity (KW)**	**Capacity**
Settler	4,300			5m^3/hr
Belt dryer[12]	29,999	15	12	400kg/hr
Mix tank	105,333	0.536	8.24	83.7m^3
Heat exchanger	4,030	0.00302		
Pressure filter	59,000	0.184	2.82	
Centrifuge	316,000	2.98	45.8	32.7m^3/hr

Note: The equipment purchase costs are referenced from *Biosteam*[13].

Supplementary Table 6. Labor Cost Breakdown

Position	2024 Salary ($)
Plant Manager*	77,809
Plant Engineer (civil) *	41,025
Plant Engineer (environmental) *	41,680
Maintenance Tech	42,345
Lab Manager*	29,641
Lab Technician	42,345
Shift Supervisor	50,814
Module operator - Production	26,910
Module operator - Dewatering	38,590
Clerks & Secretaries	38,110
Total Salaries	410,214
Labor Burden (90%)	369,193

Note: * Labor requirements are assumed to have a 50% allocation through integration with the wastewater treatment facility, as duties are shared between the wastewater treatment and algae operations.

Supplementary Table 7. Total Capital Cost

Cost ($)	Open Pond	PBR
**Storage**	62,532	62,532
**Open pond**	75,675	15,955,515
**Separation and dryer**	127,998	2,685,760
**Extraction**	681,630	681,630
**Piping & Control system**	88,530	1,932,291
**Total purchased cost**	1,036,365	21,317,728
**Balance of Plant**	310,910	6,395,318
**Lang factor**	3.0	3.0
**Total capital cost**	4,041,824	83,139,138

Supplementary Table 8. Key factors of Sensitivity Analysis for Minimum Selling Price of Bioplastics ($/kg)

Factor	Scenario	Worse	Base	Better
Bioplastic yield (0.5; 1x; 1.5x)	Pond	4.03	3.58	3.43
Production target (300t; 500t; 1000t)	Pond	4.57	3.58	2.93
Medium utilization (1.2x; 1x; 0.8X)	Pond	3.79	3.58	3.37
Electricity cost ($0.1; $0.068; $0.03/kWh)	Pond	3.58	3.58	3.58
Carbon credit ($0; $0; $120/ton)	Pond	3.58	3.58	3.52
Hydraulic retention time (10d; 8d; 3d)	Pond	3.69	3.58	3.30
Bioplastic yield (0.5; 1x; 1.5x)	PBR	37.39	30.68	26.20
Production target (300t; 500t; 1000t)	PBR	31.73	30.68	29.97
Medium utilization (1.2x; 1x; 0.8X)	PBR	30.89	30.68	30.47
Electricity cost ($0.1; $0.068; $0.03/kWh)	PBR	30.79	30.68	30.54
Carbon credit ($0; $0; $120/ton)	PBR	30.68	30.68	30.62
Hydraulic retention time (10d; 8d; 3d)	PBR	37.39	30.68	13.89

Supplementary References

1. Ou, L. *et al.* Utilizing high-purity carbon dioxide sources for algae cultivation and biofuel production in the United States: Opportunities and challenges. *Journal of Cleaner Production* **321**, 128779 (2021).
2. Singh, U., Banerjee, S. & Hawkins, T. R. Implications of CO2 Sourcing on the Life-Cycle Greenhouse Gas Emissions and Costs of Algae Biofuels. *ACS Sustainable Chem. Eng.* **11**, 14435–14444 (2023).
3. Álvarez-González, A. *et al.* Environmental and economic benefits of using microalgae grown in wastewater as biofertilizer for lettuce cultivation. *Bioresource Technology* **424**, 132230 (2025).
4. Vinasco, J. P. S. GHG emissions from algal facultative ponds under tropical conditions. in *Greenhouse Gas Emissions from Ecotechnologies for Wastewater Treatment* (CRC Press, 2021).
5. Babich, O. *et al.* Fermentation of micro- and macroalgae as a way to produce value-added products. *Biotechnology Reports* **41**, e00827 (2024).
6. Heimersson, S., Morgan-Sagastume, F., Peters, G. M., Werker, A. & Svanström, M. Methodological issues in life cycle assessment of mixed-culture polyhydroxyalkanoate production utilising waste as feedstock. *New Biotechnology* **31**, 383–393 (2014).
7. Nizamuddin, S., Baloch, A. J., Chen, C., Arif, M. & Mubarak, N. M. Bio-based plastics, biodegradable plastics, and compostable plastics: biodegradation mechanism, biodegradability standards and environmental stratagem. *International Biodeterioration & Biodegradation* **195**, 105887 (2024).
8. Alibaba. Alibaba Chemical Price. https://www.alibaba.com (2025).

9. Watkins, J. *et al.* Techno-economic analysis of bioplastic and biofuel production from a high-ash microalgae biofilm cultivated in effluent from a municipal anaerobic digester. *Algal Research* **84**, 103774 (2024).

10. Maxwell, C. Cost Indices – Towering Skills. *Cost Indices* https://toweringskills.com/financial-analysis/cost-indices/ (2020).

11. Nobre, M. L. *et al.* Techno-economic analysis of a circular microalgal approach for enhanced wastewater treatment and resource recovery in Northern Portugal. *Journal of Cleaner Production* **434**, 140389 (2024).

12. Alibaba. Alibaba Dryer Price. https://www.alibaba.com/product-detail/Belt-Type-Continue-Seaweed-Dryer-Machine_10000029775412.html (2025).

13. Cortes-Peña, Y., Kumar, D., Singh, V. & Guest, J. S. BioSTEAM: A Fast and Flexible Platform for the Design, Simulation, and Techno-Economic Analysis of Biorefineries under Uncertainty. *ACS Sustainable Chem. Eng.* **8**, 3302–3310 (2020).
