# Supplementary material for: Remediation and upcycling of microplastics by algae with wastewater nutrient removal and bioproduction potential
Source: Nat Commun. 2025 Dec 22;16:11570. doi: 10.1038/s41467-025-67543-5 (PMC12748894; doi:10.1038/s41467-025-67543-5)
Supplement: Supplementary file 2 — Description of Additional Supplementary Files [file 41467_2025_67543_MOESM2_ESM.pdf]

Description of Additional Supplementary Files

File name: Supplementary Data 1

Description: Environmental impacts of RUMBA under different scenarios.
