# Supplementary material for: Remediation and upcycling of microplastics by algae with wastewater nutrient removal and bioproduction potential
Source: Nat Commun. 2025 Dec 22;16:11570. doi: 10.1038/s41467-025-67543-5 (PMC12748894; doi:10.1038/s41467-025-67543-5)
Supplement: Supplementary file 4 — Reporting Summary [file 41467_2025_67543_MOESM4_ESM.pdf]

Corresponding author(s): Susie Y. Dai, Joshua S. Yuan

Last updated by author(s): Oct 28, 2025

Reporting Summary

Nature Portfolio wishes to improve the reproducibility of the work that we publish. This form provides structure for consistency and transparency in reporting. For further information on Nature Portfolio policies, see our Editorial Policies and the Editorial Policy Checklist.

Statistics

For all statistical analyses, confirm that the following items are present in the figure legend, table legend, main text, or Methods section.

n/a	Confirmed	
☐	☒	The exact sample size (*n*) for each experimental group/condition, given as a discrete number and unit of measurement
☒	☐	A statement on whether measurements were taken from distinct samples or whether the same sample was measured repeatedly
☐	☒	The statistical test(s) used AND whether they are one- or two-sided<br>*Only common tests should be described solely by name; describe more complex techniques in the Methods section.*
☒	☐	A description of all covariates tested
☒	☐	A description of any assumptions or corrections, such as tests of normality and adjustment for multiple comparisons
☐	☒	A full description of the statistical parameters including central tendency (e.g. means) or other basic estimates (e.g. regression coefficient) AND variation (e.g. standard deviation) or associated estimates of uncertainty (e.g. confidence intervals)
☐	☒	For null hypothesis testing, the test statistic (e.g. *F*, *t*, *r*) with confidence intervals, effect sizes, degrees of freedom and *P* value noted<br>*Give P values as exact values whenever suitable.*
☒	☐	For Bayesian analysis, information on the choice of priors and Markov chain Monte Carlo settings
☒	☐	For hierarchical and complex designs, identification of the appropriate level for tests and full reporting of outcomes
☒	☐	Estimates of effect sizes (e.g. Cohen's *d*, Pearson's *r*), indicating how they were calculated

Our web collection on statistics for biologists contains articles on many of the points above.

Software and code

Policy information about availability of computer code

Data collection	The OD and Turbidity measurements were performed with SpectraMax® iD5 (Molecular Devices) using software SoftMax Pro 7. The TGA analysis was carried out with Pyris 1 TGA using software PYRIS Software version 5.00.02. The mechanical performance testing was carried out with 100 Series Modular Universal Test Machines using software Xy version 4.00.10. The zeta potentials of cyanobacterial cells and PS microplastics were measured using a Zetasizer Advance Pro (Malvern Panalytical).
Data analysis	The calculation for mechanical testing was carried out with the Origin software (OriginPro 2021, US). All other calculations were carried out using Microsoft Excel worksheet.

For manuscripts utilizing custom algorithms or software that are central to the research but not yet described in published literature, software must be made available to editors and reviewers. We strongly encourage code deposition in a community repository (e.g. GitHub). See the Nature Portfolio guidelines for submitting code & software for further information.

Data

Policy information about availability of data

All manuscripts must include a data availability statement. This statement should provide the following information, where applicable:

- Accession codes, unique identifiers, or web links for publicly available datasets
- A description of any restrictions on data availability
- For clinical datasets or third party data, please ensure that the statement adheres to our policy

All data used to generate main text and Supplementary Figures is available from the corresponding author upon reasonable request. The corresponding author will respond to all reasonable requests within 15 business days. All data needed to evaluate the conclusions in the paper are presented in the paper and the supplementary materials. Source data are provided with this paper.

Research involving human participants, their data, or biological material

Policy information about studies with human participants or human data. See also policy information about sex, gender (identity/presentation), and sexual orientation and race, ethnicity and racism.

Reporting on sex and gender	N.A.
Reporting on race, ethnicity, or other socially relevant groupings	N.A.
Population characteristics	N.A.
Recruitment	N.A.
Ethics oversight	N.A.

Note that full information on the approval of the study protocol must also be provided in the manuscript.

Field-specific reporting

Please select the one below that is the best fit for your research. If you are not sure, read the appropriate sections before making your selection.

☐ Life sciences ☐ Behavioural & social sciences ☒ Ecological, evolutionary & environmental sciences

For a reference copy of the document with all sections, see nature.com/documents/nr-reporting-summary-flat.pdf

Ecological, evolutionary & environmental sciences study design

All studies must disclose on these points even when the disclosure is negative.

Study description

This study developed efficient remediation and upcycling of microplastics by algae (RUMBA) while synergizing with plastic upcycling, wastewater treatment, and algal production to achieve sustainability, scalability, broad applicability, and enhanced value proposition. RUMBA is based on a new mechanism for microplastic removal, where a uniquely engineered cyanobacterium (or blue-green algae) strain enhances hydrophobic interaction between cell surface and microplastics, enabling cell-microplastics aggregation and removal. The platform leads to rapid and efficient microplastic removal and achieves a superior efficiency and capacity. Furthermore, we also discovered that microplastics-enriched cyanobacteria can be upcycled to produce plastic composite with unique performance. We further showcased the integration of microplastic removal with cyanobacterial photosynthetic bioproduction for CO2 utilization and potentially surface water and wastewater treatment. The study is a simple comparative experimental design and mainly involves in comparing the performance of an engineered cyanobacteria with its wildtype strain.

Research sample

Cyanobacteria Sample:
The wild-type cyanobacterium strain used in this study was Synechococcus elongatus UTEX 2973. This strain was genetically engineered to overexpress a limonene synthase enzyme derived from spearmint (Mentha spicata).

Standard Microplastics Samples:
Polystyrene microplastics were purchased from Sigma-Aldrich in three sizes: 200 nm (Product No. 69057), 500 nm (Product No. 59769), and 800 nm (Product No. 65984). Polyethylene microplastics (32–38 µm) were obtained from Cospheric (Product No. UVPMS-BV-1.00). Polyethylene terephthalate (PET) microplastics were sourced from Goodfellow (Product No. LS527790).

Environmental Microplastics Samples:
Surface water microplastics were isolated from surface water samples collected at the Texas A&M University Research Park (30.60256, -96.36066). Wastewater microplastics were isolated from influent wastewater samples collected at the Texas A&M wastewater treatment plant (0.564560, -96.370108).

	Wastewater Samples:<br>Wastewater samples used in this study were collected from the Texas A&M wastewater treatment plant. Influent samples were collected at 30.564560, -96.370108, and effluent samples were collected at 30.565693, -96.370906.
Sampling strategy	No formal calculation was performed to determine sample sizes in this study.<br><br>For environmental microplastic sampling, 200 L of wastewater or surface water was filtered to collect potential microplastic particles. This sample volume was deemed sufficient, as microplastics were successfully isolated from these samples.<br><br>For laboratory experiments, a minimum of three independent replicates were conducted to ensure the consistency and reproducibility of the results.
Data collection	All data collection procedures are detailed in the Materials and Methods section of the manuscript. The polystyrene microplastic removal experiments were conducted by BL and QL. TEM results were obtained by BL, while SEM results were recorded by QL. SRS data were collected by YZ. The microplastics and cell interaction results were documented by BL, and the mechanical measurements were performed by CH.
Timing and spatial scale	This is not relevant to this study, as it does not involve long-term environmental data collection. All data in this study were collected in a controlled laboratory setting, with no gaps between collection periods.
Data exclusions	No data were excluded.
Reproducibility	All experiments were repeated at least three times and similar results were reproduced.
Randomization	This is not relevant due to the relatively small sample sizes used in this study.
Blinding	This is not relevant due to the relatively small sample sizes used in this study.

Did the study involve field work? ☐ Yes ☒ No

Reporting for specific materials, systems and methods

We require information from authors about some types of materials, experimental systems and methods used in many studies. Here, indicate whether each material, system or method listed is relevant to your study. If you are not sure if a list item applies to your research, read the appropriate section before selecting a response.

Materials & experimental systems

n/a	Involved in the study
☒	☐ Antibodies
☒	☐ Eukaryotic cell lines
☒	☐ Palaeontology and archaeology
☒	☐ Animals and other organisms
☒	☐ Clinical data
☒	☐ Dual use research of concern
☒	☐ Plants

Methods

n/a	Involved in the study
☒	☐ ChIP-seq
☒	☐ Flow cytometry
☒	☐ MRI-based neuroimaging

Plants

Seed stocks	N.A.
Novel plant genotypes	N.A.
Authentication	N.A.
